# A Neurocognitive Framework for Human Creative Thought

**DOI:** 10.3389/fpsyg.2016.02078

**Published:** 2017-01-10

**Authors:** Arne Dietrich, Hilde Haider

**Affiliations:** ^1^Department of Psychology, American University of BeirutBeirut, Lebanon; ^2^Department of Psychology, University of CologneCologne, Germany

**Keywords:** connectionist architecture, consciousness, creativity, default network, emulation/simulation, evolutionary psychology, prediction, task set

## Abstract

We are an intensely creative species. Creativity is the fountainhead of our civilizations and a defining characteristic of what makes us human. But for all its prominence at the apex of human mental faculties, we know next to nothing about how brains generate creative ideas. With all previous attempts to tighten the screws on this vexed problem unsuccessful – right brains, divergent thinking, defocused attention, default mode network, alpha enhancement, prefrontal activation, etc. (Dietrich and Kanso, 2010) – the neuroscientific study of creativity finds itself in a theoretical arid zone that has perhaps no equal in psychology. We propose here a general framework for a fresh attack on the problem and set it out under 10 foundational concepts. Most of the ideas we favor are part and parcel of the standard conceptual toolbox of cognitive neuroscience but their combination and significance to creativity are original. By outlining, even in such broad strokes, the theoretical landscape of cognitive neuroscience as it relates to creative insights, we hope to bring into clear focus the key enabling factors that are likely to have a hand in computing ideational combinations in the brain.

## Introduction

The last half century has seen a veritable explosion of knowledge about the mind and how it works. Perhaps the single most glaring exception in this success story is creative thinking. Indeed, it is hard to think of a mental phenomenon so central to the human condition that we understand so little. Careful reviews of the recent literature on the neuroscience of creativity (Dietrich and Kanso, 2010; Sawyer, 2011; Weisberg, 2013; Yoruk and Runco, 2014) have shown that the field is heavily fragmented, with data being selectively recruited to support concepts that are theoretically incoherent and cannot do the explanatory work we require in neuroscience (Dietrich, 2015). At this point, there is not a single cognitive or neural mechanism we can rely on to explain the extraordinary creative capacities of an Einstein or a Shakespeare.

The principal reason for this situation is that all current psychometric tests used to look for creativity in the brain are based on divisions – divergent thinking, defocused attention, remote associations, for instance – that (1) are false category formations, given their exact opposites – convergent thinking, focused attention, or close associations, in this case – also precipitate creative ideas (Dietrich, 2007b) and (2) result in constructs that still consist of many separate mental processes that are distributed in the brain. For neuroimaging studies, the combination of both theoretical problems – false category formation and compound construct – makes defeat certain. Simply put, these so-called creativity tests, such as the Alternative Uses Test (AUT) that are based on the division of divergent thinking, cannot identify the cognitive or neural processes that turn “normal” thinking into creative thinking. And if you fail to isolate the subject of interest in your study, you cannot use neuroimaging to hunt for mechanisms. You just don’t know what the brain image shows (Dietrich and Kanso, 2010; Sawyer, 2011).

## Aim and Scope

The central, motivating intent of this paper is to show a possible way out of the disciplinary insolvency in which the neuroscientific study of creativity currently finds itself. There is a whole host of evidently relevant concepts that, despite being securely anchored in the bedrock of mainstream cognitive neuroscience, have so far been ignored in creativity research. As a matter of tactics, we confine ourselves in this first approach to 10 such concepts. Together they form a neurocognitive framework less intended to offer a specific set of hypotheses but rather to inform future experiments on, and theorizing about, the creative process occurring in human brains. Although the framework does intimate testable hypotheses, our general strategy at this early stage is to survey the theoretical landscape and highlight those concepts that might open altogether new avenues of research in the field of creativity.

To suitably constrain the scope of our framework further, we focus exclusively on the computation of creative insights. Creativity typically refers to a product – *something* useful, novel and surprising (Simonton, 2012). An ingenious idea is often the first step toward a creative product but this is neither necessary nor sufficient. Creative products come into existence without the incidence of an antecedent, conscious thought, and creative thoughts have no impact unless converted into an actual product. Specifically, we leave to one side steps of the creative process dealing with the implementation of a creative idea which often requires additional creative thinking. We also pass over higher-order evaluative processes, that is, those cognitive processes that assess the original idea’s merits after it manifested itself in consciousness. Those processes required for the idea’s successful execution as well as those carrying out appraisals at the explicit information-processing level are likely to engage different cognitive processes and different brain regions (Dietrich, 2004b).

To pursue the question of how brains compute creative ideas, we bring to the fore a number of well-established neuroscientific concepts whose explanatory power with respect to creative cognition has not been realized. Collectively, they sketch out the contours of a broad framework consisting of what might be called “foundational concepts” for human creativity. We present the foundational concepts under 10 headings as follows.

## The Framework’S 10 Foundational Concepts

### Evolutionary Algorithms

More than half a century ago, Campbell (1960) proposed that creative thoughts result from the twofold Darwinian process of blind variation (BV) followed by selective retention (SR), or BVSR (see also Campbell, 1974; Popper, 1984; Simonton, 1999). A long debate on the exact parameters of the evolutionary algorithm, and especially the matter of blindness, has recently settled on a broad consensus (see Kronfeldner, 2010; Dietrich, 2015) that culture is a variational system with some coupling between variation and selection. This partial coupling means that human cultural transmission, and thus human creativity, is partially directed and thus fits, strictly speaking, neither into the rigid category requirements of Neo-Darwinian (total) blindness nor Lamarckian (total) sightedness (Richerson and Boyd, 2005; Kronfeldner, 2010). Despite this common denominator on the basic mechanism of human creativity, the two-step evolutionary rationale has been nearly universally ignored in setting up empirical protocols in neuroscience. All current psychometric measures of creativity collapse the two fundamental constituent elements of the creative process, and it is hard to imagine useful neuroimaging data from studies blending variation with selection, given that both likely engage different cognitive processes and different brain areas (Dietrich, 2004b).

The understanding of creativity as a partially sighted variation-selection process should guide the search for the brain mechanisms underlying creativity. One place to start this quest are four features that distinguish evolutionary algorithms occurring in brains from those transforming nature, as it is these four features that can be linked to a neural mechanism (Dietrich and Haider, 2015). They are: (1) cognitive coupling providing degrees of sightedness, (2) establishment of fitness values for hypothetical selection processes, (3) cognitive scaffolding for multistep thought trials, and (4) the experiences of foresight and intention.

We have proposed that the main neural mechanism that enables the cognitive coupling of variation to selection is the brain’s prediction machinery (Dietrich, 2015; Dietrich and Haider, 2015). In computational terms, this results in advanced heuristic algorithms that can boost the effectiveness of the blind, *ex-post-facto* search algorithm of the biosphere by orders of magnitude. This partial sightedness must necessarily be driven via predictive processes.

For the mind’s second adaptation, we first need to describe a complication inherent in thought trials. Evolutionary algorithms require a fitness function. In the biosphere, this is done by causal factors in the environment; that is, selection occurs in the real world, on individuals made flesh. But in simulations, or hypothetical thought trials, the selection process depends on merit criteria that must also be modeled. On what basis is this done? Since the very essence of creativity is to go into uncharted territory, how do we know what *would be* adaptive in that unknown topography.

A third adaptation that enhances the basic evolutionary algorithm is scaffolding. In nature, every variation-selection cycle in a species’ trajectory is actualized and must, in its own right, be a viable form. The basic move in Darwinian evolution, in other words, is to generate-and-field-test. Brains, on the other hand, can short-circuit instantiation and breed multiple generations in a hypothetical manner. The basic move, then, becomes to generate-and-hypothesis-test. This produces a striking effect. Because some designs require elements that cannot be realized without a temporary scaffold, a mechanism that includes an instant pay-off requirement, such as biological evolution, can also not build them. What scaffolding permits is that trajectories through the infosphere can bypass impossible intermediates. The benefit is a plethora of higher-order, discontinuous design solutions. Cognitive scaffolding also has important implications for the debate on continuous versus discontinuous processing in insight formation.

Finally, the creative process in the biosphere is not teleological. It serves no end, and its designs are neither premeditated nor deliberately initiated in response to a perceived need. Human creators, by contrast, act on purpose; they create with intent and with an objective in mind. Although one might expect such improvements in a process that inexorably bootstraps, this argument is typically framed in cognitive psychology in terms of expert systems and often falsely considered at odds with evolutionary models of creativity.

### Predictive Processing

The second foundational concept, the assumption of predictive computation, holds that the universal principle of brain function is to generate predictions (Wolpert et al., 2003; Grush, 2004; Bar, 2007; Clark, 2013), making a perpetual variation-selection search process the brain’s default operating mode. The core idea is that for behavior to be purposeful and timely in a high-dimensional environment, we must continuously, automatically, and unconsciously generating expectations that meaningfully inform – constrain – perception and action at every turn (Wolpert et al., 1995; Llinas and Roy, 2009; Clark, 2013). Even when not engaged in a specific task, the brain actively produces predictions that anticipate future events (Moulton and Kosslyn, 2009).

The brain’s prediction machinery offers a mechanistic explanation for the complex properties of cultural evolutionary algorithms running in brains that we outlined in the previous section. Specifically, our idea is that internal representations of the emulated future, which we call ideational RPGs, or Representations of Predictive Goals, provide the neural mechanism for the four special properties of our mind’s evolutionary algorithms. They can address (1) partial sightedness or coupling, (2) the ability to set a fitness function in an unknown solution space, (3) cognitive scaffolding, and (4) the feeling of foresight and intention (Dietrich, 2015).

Finally, well-established Bayesian inference techniques could tell us how such advanced evolutionary algorithms converge on a predicted goal state or the potential creative solution (Dietrich and Haider, 2015). We think that the prediction perspective, especially when embedded into a larger evolutionary frame, offers a promising direction in our search for the creative process taking place in brains.

### No Single Place; No Single Process

In foundational concept 3, we set forth the vaudeville conception of creativity (Dietrich, 2015). The vaudeville conception of creativity is based on two fundamental notions in neuroscience: modularity and non-linearity. The brain’s functional specificity, or modularity, suggests that the recombination of bits and pieces of content into novel configurations must come from the same neural circuits that normally handle those bits and pieces of content. This must also be conceded as part of our understanding of the brain as a non-linear information processor.

The tacit assumption that has been driving creativity research, however, is the opposite. Creativity is obviously special and there must be something, somewhere, that makes it so. This way of thinking betrays the commitment to a distinct factor, an extra something – the creative bit, if you like – that’s specifically added to the plain mix to make the sparkling difference. Powered by this instinctive hunch, creativity is routinely treated as a monolithic entity and assigned to some brain network (e.g., default mode network, DMN) or associated with a particular cognitive process (e.g., divergent thinking). The fact that such conclusions are based on “creativity tests” that combine a false category formation with a compound constructs, effectively renders this research paradigm phrenology.

In our view, any global statements about creativity *per se* being located in specific brain areas or networks is devoid of meaning and would border on an outright violation of the modular conception of brain function. What the vaudeville conception of creativity does is to shift the focus from mistaking colorful brain images as a substitute for an explanation to the software side of things, that is, the cognitive and computational processes that implement variation-selection runs leading to creative thoughts.

### Network Dynamics of Global Competition

The fourth foundational concept is the brain’s connectionist architecture. It takes the conventional position that information processing – selective attention, working memory, or cognitive control – involves large-scale competition between widely distributed representations that are biased by top-down, prefrontal activity (e.g., Baars, 1988; Dehaene and Changeux, 2011).

One important element that might shed light on the computation of creative ideas is the strengthening mechanism of connectionist models, as it is this mechanism that helps transient coalitions to reach threshold levels and turn them into conscious representations. The release of dopamine from neurons in the ventral tegmental area, and their subsequent activity in prefrontal and limbic regions, is currently the main proposal for such mechanism (Schultz, 2000; Rose et al., 2010). The possibility that a dopamine signal precedes the emergence of a creative insight might inform more precise neuroscience research on creativity.

### Dual Systems

For foundational concept 5, we add one more layer of complexity to the basic connectionist platform, the view that two distinct systems for knowledge representation exist, one implicit and one explicit (Reber, 1993; Dienes and Perner, 1999). This distinction seems to matter a great deal for the urgently needed task of parsing creativity into different types that have some validity.

The explicit system is a sophisticated system that is tied to consciousness and thus capable of representing knowledge in a higher-order format. In contrast, the implicit system is inaccessible to consciousness. It is stimulus-driven and, as its information is encapsulated, it cannot form such higher-order representations (Dienes and Perner, 2002; Haider and Frensch, 2009; Haider et al., 2011). Due to these encapsulated representations, the explicit system, or any other functional system in the brain, does not know about knowledge imprinted in the implicit system. However, implicit knowledge can affect performance by, for instance, biasing our current predictions.

The differences between these two systems in terms of creative capacity have been treated elsewhere (Dietrich, 2004a, 2015). Here, we only briefly highlight some differences related to the predictive machinery of each system (Downing, 2009). Due to the implicit system’s inability to represent hypothetical future scenarios, implicit prediction is online; that is, it works only in known and currently present solution spaces. In general, the implicit system uses a stochastic process to optimize behavior, simply testing out, by trial and error, solutions to environmental contingencies (Perruchet and Vinter, 2002; Haider et al., 2011). The implicit system does use prediction – in the motor system, for instance – but can only do so for already learned actions. It cannot launch ideational RPGs into abstract and unknown solution spaces. In terms of sightedness, prediction in the implicit system only possesses (partial) sightedness for known problem spaces, a situation that does not really qualify as creativity. For explorations in terra incognita, we have proposed that the implicit system can still be creative, but this creativity must be limited to the blind algorithms in nature (Dietrich and Haider, 2015). We have also hypothesized that these features are more consistent with the flow state (Dietrich, 2015).

The game-changing advantage of explicit prediction is that the explicit system can generate ideational RPGs that can be used to gain some sightedness in unknown problem spaces. Explicit prediction can thus operate oﬄine; that is, on problems that are hypothetical and that can be solved outside real time (Grush, 2004; Moulton and Kosslyn, 2009). Ideational RPGs, in other words, internalize the selection process since the parameters of that goal state prediction work as a fitness function. With the ability to simulate a complete *internal* model, we can imagine – predict the effects of – events in uncharted territory.

### Task Sets

Foundational concepts 6 is the construct of a task set (Allport et al., 1994; Monsell, 2003; Dreisbach and Haider, 2009). A task set denotes the configuration of mental resources that goes with a task. Through instructions or schemas, it defines those aspects of the task to which we selectively attend, the features of the stimulus that are bound to certain dimensions of the response as well as the response selection. The construct was formulated in response to experiments providing evidence that switching between different tasks produces substantial performance costs (Allport and Wylie, 2000; Monsell, 2003).

We cannot perform a task until the cognitive system is properly attuned and organized. If the task changes the new task set must first be uploaded, so to speak. It is a kind of mindset containing the elements and their values that are tagged as temporarily belonging together in the network because they played a role in completing the task in the past. By facilitating, top-down, certain task-relevant cognitive operations and inhibiting others, the implementation of a task set affects the processing of all stimuli associated with that task and, by extension, of a problem space.

A task set guarantees internal stability to keep the ongoing task free from interference and disruption by other task sets (Dreisbach and Haider, 2009). At the same time, task-set activation must also allow enough flexibility for mental gear changing so that we can adjust should the context necessitate it (Neumann, 1984).

The importance of this theoretical construct to the phenomena of creativity should be immediately self-evident. The task representation governs, for instance, how we would initially approach a problem-solving task (Knoblich et al., 1999; Öllinger et al., 2013). It also maps the shape of the solution space and establishes critical search parameters. These settings are, in effect, predictions about the kinds of solutions that are likely. Moreover, task set strength determines the degree of functional fixedness, or cognitive flexibility, and the probability for remote associations.

### Task-Set Inertia

Foundational concept 7 is the related notion of task-set inertia. Task-set inertia (Allport et al., 1994) was introduced to explain an unexpected asymmetry in task-switching studies that could not be accounted for with task-set reconfiguration alone. Like task sets, it is also a concept that, as far as we know, has not yet been applied to creative thinking, despite its obvious relevance to several creativity phenomena.

Since neural networks are not on/off switches, we can expect that a strongly interacting coalition of neurons does not instantly decay back to baseline. Any disintegration phase would take time, during which a new task set would be subjected to interference from the previous one. It would seem obvious that task-set inertia, extended to creative thinking, holds precious clues for understanding incubation. The fact that the removal of a problem from conscious awareness can break the impasse that often frustrates the problem-solving process shows that the task-set coalition associated with it must continue to reverberate with purpose.

But carryover activation in the knowledge structure is unlikely to be the only mechanism here. For instance, creative insights, have a way of popping up long after we last worked on a problem and it is hard to see how transient task-set inertia could linger for days or weeks. Also, the fact that there remains a problem in need of a solution is unlikely to be embedded at the level of the knowledge structure itself. This is a type of goal representation and it should require higher-order brain regions, such as the prefrontal cortex.

One way to address these complications might involve the notion of fringe working memory (Cowan, 1999, 2005). Working memory is thought to have a focal center and a fringe, with the latter containing information that still has some conscious properties. Following a task switch, a goal representation could remain active in the fringes of working memory and continue to provide, via top-down projections, some organizational control to steer the spreading activation in the task-set coalition toward a solution. Task-set inertia and fringe working memory are concepts that would seem to provide propulsive help in understanding the mechanism that rearranges bits of information into ideational combinations while the conscious mind is otherwise applied.

### Large-Scale Networks

Foundational concept 8 relates large-scale brain networks – the central executive and the DMN, in particular – to creativity (Raichle et al., 2001; Bressler and Menon, 2010). We explore what we can, and cannot, say about them in the context of creative thinking.

The DMN is a set of neural regions that shows heightened activity during resting states as well as during a number of directed mental tasks, which led to the idea that DMN activity supports mind-wandering or moments of introspective self-talk and thought (Mason et al., 2007). More recently, the DMN is often characterized as being involved in predictive processing and the ability to simulate worlds that differ mentally, temporally, and physically from the present. It includes medial temporal lobe structures, especially the hippocampus and parahippocampal cortex, the medial parietal and lateral temporal cortices, especially the temporal-parietal junction, as well as the medial prefrontal cortex, cerebellum and thalamus (Buckner, 2012).

We have the foreboding sense that the recent proposals that link the DMN to creativity has appealed to some for the unfortunate reason that it feeds into old and misbegotten category formations about creativity, such as divergent thinking or daydreaming. But there is no reason to presume that the other, central-executive network (CEN) is not also involved in creative thinking.

The CEN is anchored in the dorsolateral prefrontal cortex and several areas of the posterior parietal cortex (Bressler and Menon, 2010). It controls executive functions and shows activity whenever, we have to focus our attention on a specific task. Indeed, activity in the CEN is inversely correlated with the DMN. They operate like two components of a flip-flop circuit; while the DMN is associated with endogenous activity, the CEN is driven by exogenous input. As might be expected, predictive processing has also been associated with this CEN (Downing, 2009; Clark, 2013).

The notion that the DMN is a proactive system implies that there must be a continuous search process taking place that reduces uncertainty even when no task is at hand. This constant anticipatory drive in the DMN during moments of passive contemplation brings it into close contact with the concept of task-set inertia and the specific phenomenon of incubation. If the DMN is active during introspective simulations of the future and, by extension, simulations of possible alternative solutions to a problem, we can assume that it also shows inertia once a problem is incubated. This possibility could inform neuroimaging research on incubation.

We are careful not to associate one network, or some kind of back-and-forth interplay between them with creativity *per se*, or divergent thinking for that matter (Beaty et al., 2016). This, we think, is just another false category formation and a version of the monolithic entity fallacy. Rather, we consider that the two processing modes, or the two core networks, support different types of creative thinking.

### The Deliberate Mode

Finally, we close by defending, under headings 9 and 10, the proposal that there are two distinct modes, or types, of creativity that emanate from the explicit system, a deliberate, top-down mode (foundational concept 9) and a spontaneous, bottom-up mode of processing (foundational concept 10; Dietrich, 2004b, 2007a). The decomposition of creativity into variation and selection aside, this deliberate-spontaneous partition of creativity, along with a third flow mode that emanates from the implicit system, is the only one that we think has empirical and theoretical support. We also suggest that a mapping of the two modes on to the CEN and DMN might provide more hypotheses for future imaging studies.

The deliberate problem-solving mode is strongly biased by top-down pathways from the prefrontal cortex so that the rearrangement of informational units has built-in predispositions that are likely constrained by biases, expectancies, schemas, and previous experiences. In other words, the search function is restricted to more commonsense solutions that are more paradigmatic and rely on more close associations. But being tied to effortful and conscious processing, the deliberate mode also enables us to bring the full toolbox of our higher-cognitive function to bear on the problem, including focusing attention, retrieval of relevant memories, and the recombination of knowledge by sustaining several representations in mind at once.

The advantage of such advanced heuristic algorithms is, of course, efficiency. But trimming the vast search space also has a drawback. The deliberate mode only works well if the solution is indeed located in the predicted area of the problem space. To quip, while the deliberate mode has the advantage of limiting the solution space, it has the disadvantage of limiting the solution space!

### The Spontaneous Mode

For foundational concept 10, we contrast the deliberate mode with novel ideas that emerge from a spontaneous problem-solving mode in which top-down influences are weakened and the search function is less directional. Although this comes with a speed and efficiency tradeoff, the spontaneous mode has the potential to chance upon more paradigm-shifting ideas or remote associations. During incubation or various altered states of consciousness, the brain shifts a problem from a deliberate to a more spontaneous mode of processing that is not controlled by intentional reasoning. This significantly weakens the supervisory, top-down biases from the prefrontal cortex that guided the effortful deliberations. The drawback, however, is that a spontaneous mode does not benefit from the higher-order and efficient forecasting ability of conscious thought.

## Conclusion

Creativity has a dubious distinction in the psychological sciences. For no other mental phenomenon so central to the human condition do we know so little as to how the brain does it. Reviews of the existing literature (e.g., Dietrich and Kanso, 2010; Sawyer, 2011) have shown that the field is heavily fragmented and its neuroscientific findings are invalidated by false category formations and compound constructs. The aim of the present paper was to suggest alternative ways to attack the problem.

Our framework of human creative thought consists of 10 foundational concepts organized into 10 separate headings. The ideas we favor are all part and parcel of cognitive psychology and neuroscience: evolutionary algorithms, predictive representations, distributed processing, connectionist architecture, explicit-implicit distinction, task set, task-set-inertia, large-scale networks, and top-down vs. bottom-up processing. However, their significance to creativity, especially the crossties we developed here among them, is original. Together they form a neurocognitive framework that provides a fresh attack on the possible mechanisms that compute ideational combinations in the brain.

As a matter of tactics, we limited ourselves to those concepts that we think hold the greatest potential for progress. The framework is not intended to be complete. But for our purposes, the degree of completeness is not important. So long as it is agreed that the combination of concepts we bring to the fore are fundamental to creative cognition and possess eminent explanatory power that has not been realized. We hope that our framework helps revitalize research on an issue that defines our humanity.

## Author Contributions

All authors listed, have made substantial, direct and intellectual contribution to the work, and approved it for publication.

## Conflict of Interest Statement

The authors declare that the research was conducted in the absence of any commercial or financial relationships that could be construed as a potential conflict of interest.
